# The Health and Welfare of Rabbits as Indicated by Post-Mortem Findings at the Slaughterhouse

**DOI:** 10.3390/ani11030659

**Published:** 2021-03-02

**Authors:** Lenka Valkova, Vladimir Vecerek, Eva Voslarova, Veronika Zavrelova, Francesca Conte, Zbynek Semerad

**Affiliations:** 1 Department of Animal Protection and Welfare and Veterinary Public Health, Faculty of Veterinary Hygiene and Ecology, University of Veterinary and Pharmaceutical Sciences Brno, 612 42 Brno, Czech Republic; H20334@vfu.cz (L.V.); vecerekv@vfu.cz (V.V.); simovav@vfu.cz (V.Z.); 2 Department of Veterinary Sciences, University of Messina, 981 68 Messina, Italy; francesca.conte@unime.it; 3 Central Veterinary Administration of the State Veterinary Administration, 120 00 Prague, Czech Republic; z.semerad@svscr.cz

**Keywords:** rabbit, slaughter, veterinary inspection, health, welfare

## Abstract

**Simple Summary:**

Veterinary inspection at the slaughterhouse plays an important role in the surveillance system for animal health and welfare. The study focused on the quantification and identification of pathological findings in rabbits slaughtered at the slaughterhouses in the Czech Republic in the period from 2010 to 2019. The ratio of the number of pathological findings to the total number of rabbits slaughtered was 0.0214, i.e., for every hundred rabbits, 2.14 findings were made documenting the impairment of health and/or welfare to an extent leading to pathological changes detected during the post-mortem inspection of the rabbits at the slaughterhouse. The pathological findings that occurred most often were those on the limbs (0.84%), trunk (0.71%), kidneys (0.17%), and liver (0.05%), along with generalized changes (0.37%). The results show that findings on the limbs and trunk were dominated by findings of traumatic origin. Findings in the kidneys and liver were most often of a chronic nature. Findings of abscesses were most frequent among generalized findings.

**Abstract:**

The aim of the study was to assess post-mortem findings according to their localization and the nature of damage and to assess the standard of health and welfare of farmed rabbits on the basis of these findings. A total of 40,206 pathological findings were recorded in 1,876,929 rabbits slaughtered at slaughterhouses in the Czech Republic in the period from 2010 to 2019. Pathological findings on the limbs (0.84%), the trunk (0.71%), the kidneys (0.17%), and the liver (0.05%), along with generalized changes (0.37%), occurred most frequently. Findings of traumatic origin dominated among findings on the limbs and trunk, which indicates the inappropriate housing and handling rabbits on farms and during transport. Findings in the kidneys and liver were most often of a chronic nature having an evident correlation with the diet of intensively fed rabbits, with shortcomings in the diet having an impact on the parenchyma with chronic manifestations in the liver and kidneys. Among the generalized findings, multiple abscesses, which were probably associated with the infection of injuries occurring during fattening, and emaciation resulting from current husbandry practices, leading to insufficient feed intake or the development of disease in some individuals, predominated.

## 1. Introduction

Veterinary inspection of animals and their carcasses at the slaughterhouse is the most widely used tool in veterinary supervision and the one that has been practiced the longest. The primary purpose of these inspections is to identify animals whose meat and organs are not suitable for human consumption and to eliminate them from the food chain. However, veterinary inspection is also of enormous importance from the viewpoint of epizootiology and checks on the health of farm animals. To date, the potential of veterinary inspection in identifying and reflecting farm animal welfare as a topical issue is an aspect that has not been fully appreciated and one that remains underutilized [1,2]. The fact that all food animals must undergo veterinary inspections makes slaughterhouses the ideal place for the collection of comprehensive information. This presents a unique opportunity for improving the surveillance of health risks from the viewpoint of both humans and animals and for effectively monitoring the welfare of livestock animals [1]. Welfare indicators from which the physical condition of rabbits may be inferred can be monitored far more easily and more precisely during the post-mortem examination of carcasses than they can on live animals on farms. This relates primarily to various injuries, bruises, scratches, abscesses, and dermatitides. Findings of this kind represent a source of information on the conditions prevailing during the course of the transport of rabbits to the slaughterhouse (acute injuries) and on the farm of origin (chronic processes) [3]. The presence of fresh injuries testifies to the standard of welfare during transport, the lairage at the slaughterhouse, stunning, and slaughter itself [4]. Any increased frequency and severity of such injuries is evidence of misconduct on the part of the transporter or the slaughterhouse operator. Thorough analysis of the nature and frequency of pathoanatomical findings may be the foundation for a ruling as to whether corrective measures need to be applied and what any such specific measures will consist of.

Despite the fact that inspection reports (particularly reports on post-mortem inspections) provide a large amount of information, systematic use of the potential this offers is still not made in the majority of countries [1]. The scientific literature is likewise relatively sparse as far as the collection and analysis of data relating to pathological findings in rabbits slaughtered at the slaughterhouse is concerned. Just a few studies have been devoted to the identification and quantification of pathoanatomical findings leading to the condemnation of rabbit carcasses at the slaughterhouse [5,6,7,8]. However, differences in the numbers of studied animals and the length of the monitoring period represent a problem preventing valid comparison. An exception in terms of scope is the long-term Polish study that states that 0.48% of all rabbit carcasses examined at Polish slaughterhouses in the years 2010–2018 were declared unfit for human consumption. The most frequent reasons for condemnation were sepsis and pyaemia, emaciation, disease of the upper respiratory tract, and coccidiosis. The occurrence of coccidiosis and other parasitic diseases fell during the monitored period, although an increase was seen in the number of cases of sepsis [8].

The studies that have monitored the health of rabbits on farms indicate that two main health problems typically occur in rabbits: respiratory syndrome (predominantly in adult individuals) and digestive syndrome (more frequently in young rabbits). Diseases of the digestive tract are the main cause of morbidity and mortality in broiler rabbits. For example, the widely distributed pathogenic bacterium *Clostridium perfringens* is a frequent agent of intestinal disease on farms [9]. Other pathological processes affecting rabbits on commercial farms include subcutaneous abscesses [7], gastrointestinal parasitoses [10], alopecia [11], and nephritis (the causal agents *Toxoplasma gondii* and *Encephalitozoon cuniculi*) [5].

This study aimed to assess characteristic findings made during the veterinary inspection of rabbits slaughtered at slaughterhouses in the Czech Republic in the period from 2010 to 2019 according to their localization and the nature of damage, and to assess the level of health and welfare of farmed rabbits on the basis of these findings.

## 2. Materials and Methods

The health and welfare of farmed rabbits were investigated in 1,876,929 rabbits slaughtered at nine slaughterhouses in the Czech Republic in the period from 2010 to 2019. The rabbits came from 80 farms, of which one farmer is entirely dominant. The production of this farmer accounts for more than 50% of total rabbit production in the Czech Republic and amounts to around 130,000 rabbits annually. The production of another 19 farmers ranges from 1000 to 10,000 rabbits a year, the annual production of another 21 farmers ranges from 100 to 1000 rabbits, while 39 are small farmers with a production of up to 100 rabbits a year. On farms, the rabbits were housed in cages and fed with pellets. The transport of rabbits from farms to slaughterhouses was carried out by authorized transporters by means of road transport using transport containers and trucks specifically designed and approved for the transportation of rabbits. In compliance with the law, feed withdrawal before slaughter did not exceed 12 h, including transportation and lairage. The majority of rabbits (88%) were transported for less than 300 km (63% of rabbits for less than 100 km), and no journey exceeded eight hours. At all slaughterhouses, rabbits were stunned by using head-only electrical stunning. Restraining was manual; the head of the rabbit was positioned in wall-mounted V-shaped electrodes that were firmly pushed between the back of the eyes and the base of the ears to span the brain. Rabbits were stunned individually and shackled, and neck cutting was performed immediately by making an incision ventrally, behind the mandibles.

The overall level of the health of rabbits slaughtered at the slaughterhouses was monitored on the basis of calculation of the ratio of the number of pathological findings to the number of rabbits slaughtered at the slaughterhouses. Data on the results of post-mortem veterinary inspections carried out at the Czech slaughterhouses were obtained retrospectively from the information system of the State Veterinary Administration of the Czech Republic.

The ratio of the number of findings in individual organs and tissues (liver, stomach, intestines, lungs, heart, spleen, reproductive organs, central nervous system (CNS) and nerve tissue, skin, head, trunk, limbs, generalized changes, other unclassified changes) to the number of rabbits slaughtered at the slaughterhouses was calculated in order to determine the organs and tissues in slaughtered rabbits most affected from the viewpoint of health.

The number of findings of an acute nature, findings of a chronic nature, parasitic findings, findings of traumatic origin, generalized findings, and other findings was monitored to determine the nature of any changes. Pathological changes associated with short-term inflammatory processes in the organism were included among acute findings. In individual organs, this involved findings taking in significant hyperemia, the presence of hemorrhages, edemas or enlarged organs, and the presence of catarrhal, fibrinous, hemorrhagic or purulent exudation, etc. Pathological changes associated with longer-lasting inflammatory processes and functional or morphological changes to tissues and organs were included among chronic findings. In individual organs, this primarily involved findings taking in changes to the original structure of parenchyma tissue, during which the proliferation of connective tissue and the formation of ligament scarring and concretions occur, as well as organ shrinkage and toughness, roughening of the structure of the surface of mucous membranes and serous surfaces, the presence of post-inflammatory pseudocysts, the presence of cysts or abscesses, calcification, etc. Traumatic findings point to the standard of the welfare of the slaughtered animals and arise immediately before slaughter (acute) or at a previous time (chronic), for which reason they were assessed separately and included open wounds at various stages of healing, hematomas in the hypodermis and muscle tissue, contusions, dislocations and fractures (open and closed), and hemorrhages and ruptures in internal organs arising as the result of inappropriate handling or direct damage caused by means of technology, transport, or fighting between animals. Changes indicating the invasion, migration, and pathological processes of parasites in the organism of the host, primarily changes pointing to the presence of coccidia in the liver and intestines, the presence of cysticerci, and perhaps other parasites, were included among parasitic findings. Inadequate development, emaciation, ascites, multiple abscesses, tumors, and other generalized changes were included among generalized changes. Other changes that could not be included in the above categories in view of the nature of the findings, such as non-inflammatory changes and tumors on the skin, jaundice, pathological pregnancy, sexual developmental defects, and other generalized or pathological organ findings that could not be included in the monitored groups of findings were included among other findings.

In order to assess the nature of the organs and tissues most affected (liver, kidneys, trunk, limbs), the nature of these changes was also assessed directly in these organs and tissues (acute, chronic, parasitic, traumatic, other).

The long-term level of the health and welfare of the rabbits slaughtered at slaughterhouses was derived from the results obtained.

The results were statistically evaluated with the program Unistat 6.5 for Excel (Unistat Ltd., London, UK). A Chi-square test was used for statistical comparison of the frequency of findings within the individual categories [12] for assessment of statistical significance in a 2 × 2 contingency table. Yates’ correction was used on frequencies exceeding 5. Fisher’s exact test was used at frequencies lower than 5 [12].

## 3. Results

A total of 40,206 pathological findings were recorded in 1,876,929 rabbits slaughtered at slaughterhouses in the Czech Republic in the period from 2010 to 2019. Therefore, the ratio of the number of pathological findings to the total number of rabbits slaughtered at slaughterhouses was 0.0214 (2.14%).

The numbers of pathological findings according to their localization in rabbits slaughtered at slaughterhouses are given in Table 1.

The pathological findings that occurred most often were those on the limbs (0.84%), trunk (0.71%), kidneys (0.17%), and liver (0.05%), along with generalized changes (0.37%). The differences in the occurrence of pathological changes between the given categories were statistically highly significant (*p* ˂ 0.01).

The distribution of findings according to their nature and/or the origin of pathological changes in rabbits slaughtered at the slaughterhouse is given in Table 2. The differences in the occurrence of changes were statistically highly significant between all categories (*p* ˂ 0.01). The largest number of findings was those of traumatic origin (1.518%). Other categories were detected in less than 1% of cases.

In view of the fact that findings occurred most often on the limbs and trunk, in addition to generalized changes, followed by the kidneys and liver, we also devoted particular attention to the detailed nature and origin of findings in these organs and tissues.

From the viewpoint of assessment of the occurrence of findings on the individual parts of the body, the most numerous findings were on the limbs and trunk. A division of findings on the limbs and trunk according to the nature and/or origin of these changes is given in Table 3. The results show that findings on the limbs were dominated by findings of traumatic origin, whose occurrence was statistically significantly (*p* ˂ 0.01) higher than findings of a chronic or acute nature. Similarly, findings on the trunk were statistically significantly (*p* ˂ 0.01) more frequently of a traumatic nature than acute and chronic findings.

From the viewpoint of assessment of the occurrence of findings in individual organs, the most numerous findings were made in the liver and kidneys. The division of findings in the kidneys and liver according to the nature and/or origin of the changes is given in Table 4. Findings in the kidneys and liver were most often of a chronic nature.

The results relating to generalized findings are given in Table 5. Findings of abscesses were most frequent among generalized findings. Emaciation was found statistically significantly (*p* ˂ 0.01) less often. The occurrence of inadequate development and ascites was entirely negligible. A statistically significant difference (*p* ˂ 0.01) was found in the occurrence of individual generalized findings.

## 4. Discussion

The ratio of the number of pathological findings to the total number of rabbits slaughtered at the slaughterhouse was 0.0214. This means that for every hundred rabbits, 2.14 findings were made documenting the impairment of health and/or welfare to an extent leading to pathological changes detected during the post-mortem inspection of the rabbits at the slaughterhouse. Methodically comparable studies have also been performed on other species of slaughter animals in the past. The only species with an occurrence of pathological findings even lower than that found in rabbits was the broiler chicken (ratio 0.016) [13]. This ratio was higher in all other studied species: in turkeys (0.101) [13], pigs (0.809) [14], and cattle (0.479) [15]. Interestingly, completely opposite results were found when comparing mortality rates in these species during their transport to slaughterhouses in the Czech Republic. The highest transport-related mortality rates were found in broiler chickens (0.37%) [16] and rabbits (0.19%) [17], with lower mortality rates found in pigs (0.07%) [18], cattle (0.02%) [19,20], ducks (0.08%) [21], and turkeys (0.15%) [22]. These results indicate that broiler chickens and rabbits with an impaired state of health die during transport to the slaughterhouse far more often than other species or, in other words, it is mainly animals in good condition that survive transport. Therefore, it is mostly animals in a good state of health that are then slaughtered, which is documented by the low numbers of pathological findings made after slaughter at the slaughterhouse in comparison with other species of meat animals. It is also possible that differing methods of transport and varying sensitivity to transport stress in different species have an effect on the proportion and condition of animals slaughtered after transport. The low number of pathological findings in broiler chickens and rabbits may also be the result of their short lifespan in comparison with other animal species reared for meat.

Little study is devoted to the quantification and identification of pathological findings in rabbits slaughtered at the slaughterhouse, which is a shame both from the scientific viewpoint and, first and foremost, from the viewpoint of veterinary care for the health and welfare of farmed rabbits. Szkucik et al. [6] studied the occurrence and nature of pathological findings in rabbits slaughtered at Polish slaughterhouses in the period from 2000 to 2010. Pathological changes or deviations were recorded in 4.94% of carcasses, with 1.05% of all slaughtered rabbits declared unfit for human consumption. The most common pathological finding was the presence of parasites, particularly coccidia (65.13%), although the most common reason (34.93%) for rejection from human consumption was bacterial disease (sepsis and pyemia), followed by coccidiosis (28.96%). The generalized finding recorded most often was emaciation (2%). This research was followed up in the years 2010 to 2018 by Drozd et al. [8], who detected pathological changes in just 0.48% of slaughtered rabbits. The most common reason for the condemnation of carcasses and internal organs was sepsis and pyemia, followed by emaciation, disease of the upper respiratory tract, other unspecified causes, and coccidiosis. A fall in the occurrence of parasitic disease in comparison with the preceding period was a positive trend. Our study also indicates that the occurrence of parasitic diseases is being successfully eliminated on rabbit farms. However, in contrast to the Polish study, the most serious health and welfare problems in the Czech Republic are not bacterial diseases inducing sepsis or pyemia, but findings of a traumatic nature (hematomas, contusions, dislocations, fractures, hemorrhages, ruptures, etc.). Rampin et al. [5] stated that pathological findings were observed in a mere 1% of the 59,440 rabbit carcasses subjected to post-mortem inspection at slaughterhouses in Italy. Findings of a traumatic nature did not play a dominant role in this study either, although pathological lesions were found most frequently on the surface of the body and in the digestive and urinary tracts. The most common findings were subcutaneous abscesses and nephritis, which were probably induced by the parasite *Encephalitozoon cuniculi*. A positive trend, as far as the health and welfare of rabbits on Czech farms is concerned, can be deduced from the study conducted in the years 1989 to 1994 and 1995 to 2000 that focused on classification of the carcasses of various farm animals (cattle, pigs, sheep, goats, horses, chickens, hens, turkeys, ducks, geese, and rabbits) slaughtered at slaughterhouses in the Czech Republic [23]. The relative number of carcasses approved for human consumption increased in the years 1995 to 2000 in comparison with the period 1989 to 1994 in all the studied species and categories of animals. In rabbits, this increase amounted to almost 10% (from 83.95 to 93.64%).

The rabbit is an extremely sensitive animal, for which evidence includes both the high level of mortality during transport [17] and the relatively high mortality in rabbits on farms, particularly during the post-weaning period [24,25,26]. As many as one-quarter of young rabbits die during fattening [27]. The short lifespan (fattening is completed before the impact of rearing conditions is manifested in changes to the organs) and high mortality during rearing and transport (the elimination of individuals with an impaired state of health) are reflected in the health and condition of rabbits slaughtered at the slaughterhouse. Nevertheless, in spite of the relatively low number of pathological findings made in rabbits during inspection at the slaughterhouse, certain categories of findings that display higher frequency and testify to specific impairment of health and welfare during rearing or transport can be identified. Findings on the trunk and limbs were clearly most frequent, with these being almost exclusively traumatic changes. Findings of a traumatic nature on the body and limbs are undesirable from the perspective of animal welfare. The cause may be a husbandry system causing injuries to the limbs in particular, as well as the method of catching rabbits and placing them in transport containers before transport and the method of unloading rabbits from transport containers at the slaughterhouse, during which injuries, contusions, dislocations and fractures probably occur. Wire floors are a frequent cause of bruising and injury (parakeratosis, pododermatitis), for which reason it is appropriate to cover them, at least with matting [28]. The high stocking density, leading to increased aggressiveness in rabbits, is also a problem [29]. Inadequate cage sizes restrict the possibilities for movement and natural activities and postures, which leads to abnormal development of the rabbit skeleton (deformed bones, hypoplasia of the bone tissue) [30]. Equipping cages or pens with enrichment features (chew sticks, elevated platforms, tunnels, chains, branches) has an unequivocally positive effect, as they make a significant contribution to stress reduction and act to prevent aggressive behavior and behavioral abnormalities such as the gnawing of cage wiring and stereotypies [31,32]. Rabbits given alternative housing (a lower stocking density, a floor with plastic grids) displayed a lower occurrence of injury, although they did show a higher mortality rate [33]. In addition to injuries occurring during rearing, further injuries (of an acute nature) also occur in connection with transport to the slaughterhouse. Handling the animals during loading and unloading is in itself a risk factor as far as the occurrence of injuries and mortality during transport is concerned. This risk is increased if handling is inconsiderate and if a large number of animals are transported within a single shipment. The cause lies in the human factor, as staff become less careful when handling a large number of animals [34]. The staff’s relationship to animals plays a key role during the handling of slaughter rabbits [35]. Rough treatment leads to traumatic lesions such as bruises, abrasions, contusions, and fractures [36]. Pathological findings of a traumatic nature in our study correspond to the high mortality rate during transport to the slaughterhouse published by Voslarova et al. [17].

Pathological findings were made most often in the kidneys and liver during the examination of organs. These were almost exclusively chronic findings in the kidneys (99.9%), and chronic findings (91.5%) also markedly exceeded acute and parasitic findings in the liver. Similarly, Rampin et al. [5] found pathological changes primarily in the digestive and urinary tracts during their examination of rabbits at the slaughterhouse in Italy. Chronic changes in the kidneys and liver are probably caused by an imbalance in the diet in relation to the needs of certain individuals during intensive fattening, and this imbalance impacts the parenchyma (the liver and kidneys). The proper functioning of the digestive system, and thereby the entire organism, is influenced not merely by the composition of the ration and the appropriate ratio of nutrients, but also by the amount of feed received, the method of feed processing, and the structure and size of its individual particles. Research results indicate that reducing the content of starch and protein in favor of fiber has beneficial effects on the digestive process and fermentation in the appendix, while preserving parameters of yield, and it leads to a decrease in the number of health problems and mortality in rabbits [37,38]. Rabbit nutrition has been studied in detail, particularly under the pressure of epizootic enteropathy, and it now plays a dominant role in the prevention of health problems [39,40]. The prevention of digestive problems and subsequent impairment of the metabolism and organ damage lie in the correct ration composition—a higher proportion of digestible and indigestible fiber (crude fiber 14–18%) [41], a lower content of starch (under 14%) [42] and protein (15–16%) [43], and possibly supplementation with suitable plant additives [44]. Restricting feed during the post-weaning period is also an effective measure [45,46].

In comparison with foreign studies [5,6,8], a surprisingly low incidence of parasitic findings in the liver was observed in the rabbits, which may testify to the containment of hepatic coccidiosis on farms. The containment of intestinal coccidiosis in the fattening of rabbits is also confirmed by the zero incidence of findings in the intestines. However, Szkucik et al. [10] draw attention to the fact that even rabbit carcasses that were approved for human consumption during post-mortem veterinary inspection need not necessarily be free of parasites. In the period from 2007 to 2011, Polish scientists examined intestines and livers obtained from carcasses that had undergone veterinary post-mortem inspection and been approved for human consumption. Parasitological tests on samples taken from the intestines and liver of rabbits slaughtered in the years 2007 to 2011 revealed the presence of gastrointestinal parasites in almost 80% of cases. The occurrence of coccidia (78.83%) was recorded most often in the internal organs of rabbits, followed by nematodes (16.42%), *Cysticercus pisiformis* (4.74%), and the tapeworm *Mosgovoyia pectinata* (0.72%) [10].

Generalized changes were also recorded to a great extent among the studied categories of findings. The great majority of these were findings of multiple abscesses (84.5%) and emaciation (14.9%). Subcutaneous abscesses are a frequent health problem on commercial rabbit farms [5]. They may be localized all over the body, although they appear most frequently on the hind legs and in the neck area. They are generally the result of the infection of injuries occurring during the course of fattening. Abscesses form most often following mutual biting or fighting between individuals housed together. *Pasteurella* spp. (59.3%) and *Staphylococcus aureus* (25.9%) are isolated most frequently during microbiological analysis [7]. There are a large number of causes of emaciation, which are mostly associated with some other pathological condition such as infections of the respiratory tract, gastroenteritis, nephritis, and hepatic and intestinal coccidiosis. Emaciation may also be the result of competitive relationships associated with access to feed during rearing preventing certain individuals from a sufficient intake of nutrients and subsequently leading in the long term to emaciation. In both previous Polish studies [6,8], emaciation was one of the most frequent reasons for the rejection of carcasses from human consumption during the slaughterhouse inspections of rabbits. Then, this is a topical problem that must be resolved at the level of modification of the husbandry and hygiene on rabbit farms.

## 5. Conclusions

The numbers of pathological findings made during the examination of rabbits at the slaughterhouses are lower in comparison with other species of animal raised for meat. This may mean either that rabbits are in good condition overall or merely the fact that it is largely rabbits in good condition that reach the slaughterhouse because those in worse condition have already died during rearing or transport, as has been indicated by previous studies documenting a high mortality rate in rabbits. Traumatic findings on the limbs and trunk, which were the most common findings made during examination at the slaughterhouses, would also correspond to an unsatisfactory level of welfare on farms and during transport. The organs most affected from the viewpoint of the number of pathological findings were the liver and kidneys. Chronic findings predominated in both cases, indicating that they originated on the farm and have an evident correlation with the nutrition of intensively fed rabbits, with nutritional shortcomings having an impact on the parenchyma with chronic manifestations in the liver and kidneys. The low level of occurrence of findings of parasitic origin in the liver and the zero findings in the intestines is positive, testifying to the management of rabbit coccidiosis during rearing. Generalized findings were dominated by multiple abscesses, which were probably associated with the infection of injuries occurring during the course of fattening, and emaciation resulting from current rearing technology leading either to certain individuals receiving an insufficient intake of feed or to the development of disease.

## Figures and Tables

**Table 1:** Occurrence of pathological changes to organs and tissues found in rabbits (n = 1,876,929) after slaughter at the slaughterhouse.

Organs and Tissues	Pathological Changes	
	Number	%
Liver	862	0.046
Stomach	0	0.000
Intestines	0	0.000
Lungs	89	0.005
Heart	0	0.000
Spleen	3	0.000
Reproductive organs	0	0.000
Kidneys	3135	0.167
Nerve tissue (CNS, spinal cord)	15	0.001
Skin	0	0.000
Head	29	0.002
Trunk	13,261	0.706
Limbs	15,717	0.837
Generalized changes	6918	0.369
Other changes	179	0.010

CNS = central nervous system.

**Table 2:** The nature and/or origin of pathological changes found in rabbits (n = 1,876,929) after slaughter at the slaughterhouse.

Nature and/or Origin of Changes	Pathological Changes	
	Number	%
Acute	277	0.015 ^d^
Chronic	4305	0.229 ^c^
Parasitic	73	0.004 ^f^
Traumatic	28,500	1.518 ^a^
Generalized	6918	0.369 ^b^
Other	134	0.007 ^e^

^a–f^ percentages lacking a common superscript differ (*p* ˂ 0.01).

**Table 3:** The nature and/or origin of pathological changes on the trunk and limbs found in rabbits (n = 1,876,929) after slaughter at the slaughterhouse.

Nature and/or Origin of Changes	Pathological Changes			
	Trunk		Limbs	
	Number	%	Number	%
Acute	168	0.009 ^c^	60	0.003 ^b^
Chronic	239	0.013 ^b^	13	0.001 ^c^
Traumatic	12,855	0.685 ^a^	15,645	0.834 ^a^

^a–c^ percentages in the same column lacking a common superscript differ (*p* ˂ 0.01).

**Table 4:** The nature and/or origin of pathological changes in the kidneys and liver found in rabbits (n = 1,876,929) after slaughter at the slaughterhouse.

Nature and/or Origin of Changes	Pathological Changes			
	Kidneys		Liver	
	Number	%	Number	%
Acute	1	0.000 ^b^	1	0.000 ^c^
Chronic	3134	0.167 ^a^	789	0.042 ^a^
Traumatic	0	0.000 ^b^	71	0.004 ^b^

^a–c^ percentages in the same column lacking a common superscript differ (*p* ˂ 0.01).

**Table 5:** The occurrence of generalized changes in rabbits (n = 1,876,929) after slaughter at the slaughterhouse.

Finding	Number	%
Inadequate development	44	0.002 ^c^
Emaciation	1027	0.055 ^b^
Abscesses	5844	0.311 ^a^
Ascites	4	0.000 ^d^

^a–d^ percentages lacking a common superscript differ (*p* ˂ 0.01).

## Data Availability

Data for analysis was obtained from the information system of the State Veterinary Administration of the Czech Republic. The datasets generated and analyzed in the current study are available from the corresponding author on reasonable request.
